# Forwarding information and communication within collaboratives: exploring the perceptions of Heads of Nursing and Allied Health Professionals

**DOI:** 10.1186/s12912-026-04490-6

**Published:** 2026-02-27

**Authors:** Filippo Vella, Maurizio Moreno Fattori, Giulia Marini, Cristina Ferrari, Federica Canzan

**Affiliations:** 1 Azienda Ospedaliera Universitaria Integrata Verona, Verona, Italy; 2 Università degli Studi di Verona Dipartimento di Scienze Umane, Verona, Italy; 3 Università degli Studi di Verona Dipartimento di Diagnostica e Sanità Pubblica, Verona, Italy

**Keywords:** Internal communication, Nursing management, Healthcare leadership, Healthcare workers, Heads of Nursing and Allied Health Professionals, Nursing leadership, Information transmission, Qualitative research

## Abstract

**Background:**

Internal communication is crucial in today’s corporate world, ensuring alignment with organizational goals and enhancing performance. However, organizational complexity and lack of standardization may hinder this effectiveness. The transmission of information from top to bottom is a critical element in ensuring effective, timely communication across hierarchical levels. The aim of this study was to analyse the process of top-down communication from Heads of Nursing and Allied Health Professionals to Healthcare Workers in hospital care units, with the specific purpose of identifying challenges and strategies to inform the design of a structured quality improvement project on internal communication.

**Methods:**

A qualitative descriptive study using a content analysis approach was conducted between June and July 2024 in a large university hospital in Northern Italy. Nineteen Heads of Nursing and Allied Health Professionals with at least two years of experience were recruited using purposive sampling. Semi-structured interviews were audio-recorded after participants provided written consent, transcribed verbatim, and analysed using content analysis by two independent researchers.

**Results:**

The analysis highlighted five main categories: (1) use of software applications for the transmission of information, (2) time management for the transmission of information, (3) use of feedback as a verification strategy, (4) contextualization of information in the care unit, and (5) the uncertainty on the effectiveness of official communication channels. The primary channels of communication are formal tools such as email and meetings, while instant messaging apps are used to disseminate urgent alerts. The effective management of time in the transmission of information is regarded as a crucial aspect. Feedback is used as a strategy to verify effective reception and understanding of information. Nevertheless, uncertainty persists about the effectiveness of official channels.

**Conclusion:**

To enhance internal communication, it is vital to strike a balance between technological efficiency and the well-being of Heads of Nursing and Allied Health Professionals, while guaranteeing the quality of care, adopting structured feedback practices, and contextualising information. An integrated approach that combines informal technologies and traditional methods is essential to address operational challenges and improve information management in the healthcare sector.

**Clinical trial number:**

Not applicable.

## Background

Communication is a fundamental element in organizational management and a strategic factor in various situations [1–4]. The effectiveness of organizational communication significantly influences an organization’s success, as it is fundamental to the management of entities [5]. Furthermore, communication encompasses vital tasks for conveying information, attitudes, ideas, feelings, planning, and decisions [6]. For communication to occur, an individual uses language to transmit a message through a channel to another individual, who interprets the message. 

In healthcare, the Heads of Nursing and Allied Health Professionals produce the message, and the staff receive it [7]. Communication between the heads of Nursing and Allied Health Professionals includes both positive and negative behaviours. Despite the inconsistencies in how communication styles are described in the literature, it is acknowledged that aspects such as respect, active listening, appropriate emotional responses, and access to sufficient information are positive. Conversely, negative behaviours include condescension, intimidation, micromanagement, contradictory communication, and an inability to convey information to employees clearly and unambiguously [7]. The limited literature in health care indicates that organizational communication facilitates the efficient exchange of patient care information among HCWs, thereby enhancing nursing effectiveness and improving overall organizational performance [8]. This type of communication is essential for high-quality healthcare delivery; without it, care quality would decline, leading to increased costs and adverse patient outcomes [9]. Direct communication between clinical nurses and their nurse managers is often the critical link for significantly impacting patient outcomes [10], resulting in greater job satisfaction, commitment, and retention for HCWs [11–14].

Conversely, in poor communication systems, high-quality patient care, health worker satisfaction, collaboration and teamwork, and leaders’ commitment to providing resources are lost. This can also hurt healthcare costs [10, 15–19]. Unfortunately, healthcare systems suffer from significant inadequacies in communication [19, 20].

The main factors contributing to poor communication included the absence or ineffectiveness of meetings, limited consultation, and irregular feedback mechanisms, which negatively affected information flow [14].

Modern healthcare organisations are complex and specialised, requiring collaboration across interprofessional teams. Within this complexity, top-down communication from leaders becomes essential to ensure clarity, alignment, and safe care delivery [21].

Communication is necessary in healthcare, whether between nurse managers and nurses, physicians, or any other combination of healthcare professionals. However, the communicative relationships between these professionals remain an underexplored area of research [22].

Recent literature highlights various analytical and communication strategies to improve healthcare team coordination and internal information flow. Grippa et al. [23] applied Social Network Analysis to assess how healthcare teams exchange information and rely on brokerage roles. Their findings show that high-performing teams exhibit dense intra-team connections and strong brokers who facilitate information flow across units, supporting timely and targeted communication. Winzer et al. [24] examined internal crisis communication in a Norwegian tertiary hospital during the COVID-19 pandemic, focusing on the role of channel selection. The study found that communication effectiveness improves significantly when high-bandwidth channels—such as in-person meetings or video calls—convey complex or urgent messages. Conversely, low-bandwidth channels (e.g., e-mail, bulletin boards) were associated with delayed understanding and reduced staff engagement.

These studies underscore the importance of integrating technical decision-making frameworks and context-sensitive communication strategies when examining internal information flows in healthcare organizations.

Much of the existing literature on healthcare communication focuses on clinical and operational dimensions, with limited attention to structured internal communication strategies that support collaboration, information dissemination, and staff engagement. Moreover, empirical evidence remains scarce on how top-down communication is enacted in everyday clinical practice by Heads of Nursing and Allied Health Professionals. Previous studies have mainly explored communication channels, crisis situations, or conceptual frameworks, often overlooking healthcare leaders’ lived experiences, decision-making processes, and practical challenges in conveying information to frontline staff.

This study addresses these gaps by exploring the role of Heads of Nursing and Allied Health Professionals in top-down communication, examining how their behaviours and strategies shape information flow and staff engagement. By capturing leaders’ perspectives in a real-world hospital setting, the findings offer practice-oriented insights to inform the development of structured interventions aimed at improving the clarity, timeliness, and effectiveness of internal communication in healthcare organizations.

Top-down communication in healthcare organizations can also be understood as a process of sensemaking, whereby leaders interpret, filter, and translate information for frontline staff in order to reduce ambiguity and support coordinated action. Sensemaking theory suggests that organizational actors actively construct meaning from complex and often incomplete information, particularly in dynamic environments such as healthcare systems [25]. In this perspective, Heads of Nursing and Allied Health Professionals function not merely as transmitters of information but as interpreters who shape how messages are understood and enacted within clinical units.

Furthermore, the selection of communication channels may be influenced by media richness considerations, as different media vary in their capacity to convey nuanced or equivocal information [26]. In highly complex contexts, richer media such as face-to-face interactions may be preferred to facilitate shared understanding. At the same time, healthcare leaders are frequently exposed to high volumes of information, raising the risk of information overload, which can impair decision-making and communication effectiveness [27]. These theoretical perspectives provide a useful lens for examining how leaders manage and forward information within collaborative healthcare environments.

Together, these theoretical perspectives suggest that internal communication in healthcare organizations is not a simple transmission process, but a complex interpretive activity shaped by organizational context, cognitive constraints, and media characteristics. This study therefore explores how Heads of Nursing and Allied Health Professionals enact top-down communication as an adaptive practice within real-world clinical environments.

## Materials and methods

### Study design and setting

A qualitative descriptive study [28] was conducted in 2024 in a University Hospital in the North-East of Italy.

The study was conducted and reported in accordance with the Consolidated Criteria for Reporting Qualitative Research (COREQ) checklist, which is provided as Supplementary Material.

The setting was a 1500-bed university hospital in Verona, Italy. The hospital has high- and low-complexity units, with 5000 healthcare providers providing direct patient care.

**Fig. 1:**
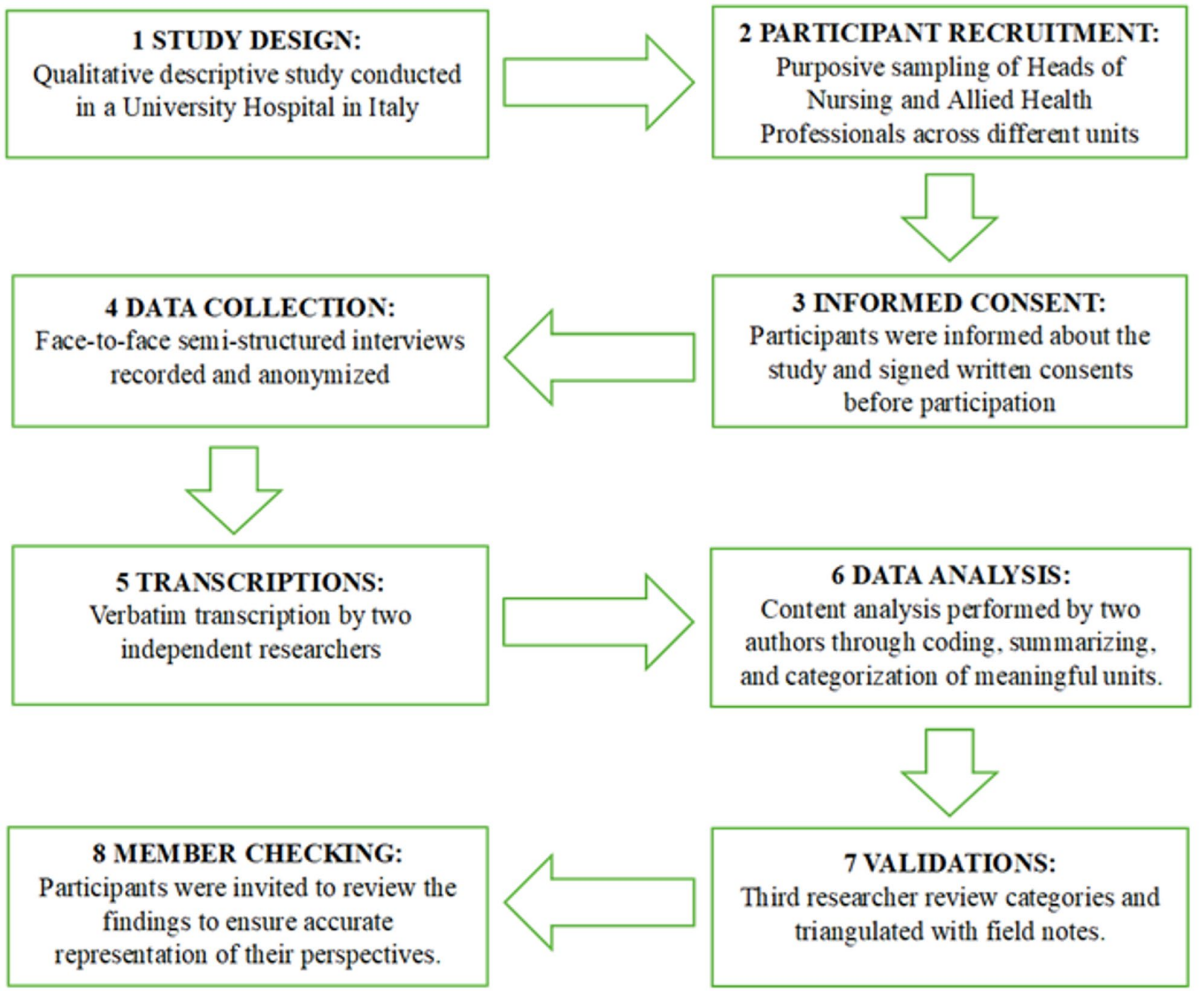
Flowchart of the methodological procedures

The University Hospital is a point of reference for the entire Veneto region, a hub, and recognized as a highly specialized national healthcare center for the activities carried out across various research, care, and training sectors. It is a hub centre [29]. There are approximately 100 Heads of Nursing and Allied Health Professionals. The overall methodological process is summarized in the following flowchart (Fig. 1), which outlines the main steps of the study.

### Sampling

Heads of Nursing and Allied Health Professionals were recruited through purposive sampling to achieve maximum variation in age, sex, education, and experience in the role, and to ensure that the participants had direct and significant experience in managing internal communication, as they were responsible for transmitting information in their Care Units.

The term “Heads of Nursing and Allied Health Professionals” is used in this study to indicate professional coordinators with managerial responsibility over healthcare staff, including nurse managers and managers of allied health disciplines (e.g., physiotherapists, midwives, laboratory technicians, and radiology technicians). The sample, therefore, included only coordinators and did not involve frontline healthcare workers.

Participants were selected from different clinical areas (medical, surgical, emergency, obstetrics-gynecology, and diagnostics) with different socio-demographic characteristics (age, gender, and professional seniority) to ensure diversity in experience and perspectives. The participants of this study met the following criteria: they were Heads of Nursing and Allied Health Professions with a minimum of two years of experience in the role, to ensure a consolidated knowledge of communication practices and challenges, and they were willing to participate in the study. Participants were excluded if they had less than two years of experience in the role, did not hold direct responsibility for internal communication within their Care Unit, or were unwilling to provide informed consent.

The sample size was not determined a priori but was based on the richness of the data collected until thematic saturation [30]. Recruitment and data collection proceeded concurrently, and interviews were continued until no new relevant categories emerged from the data. Thematic saturation was considered achieved at the 16th interview, when consecutive interviews yielded repetitive information. Three additional interviews were conducted to confirm saturation. The decision that saturation had been reached was made collectively by the research team, based on consensus and recognition that no substantial new insights were emerging.

The principal investigator contacted the Heads of Nursing and Allied Health Professionals via email, providing details of the study and the consent form. Those who indicated their willingness to participate were subsequently contacted to arrange an in-person interview, during which they provided written consent.

### Data collection

Data were collected through face-to-face semi-structured interviews between June 2024 and July 2024 following a semi-structured guide (Table 1) developed through a three-step process. First, the interview questions were developed based on a review of existing literature on internal communication in healthcare and qualitative research frameworks [31]. Second, based on these themes, a preliminary set of open-ended questions was drafted to explore perceptions, strategies, and barriers in communication between leaders and healthcare workers. Finally, the questions were reviewed by a panel of experts, including a senior nurse researcher (F.C.), who assessed content validity, clarity, and relevance. Although the interview guide was not piloted, it was carefully reviewed by experts in healthcare communication, ensuring clarity, relevance, and appropriateness of the questions.

The following data were collected at the beginning of the interview: age, gender, education, role, years of work experience, and work setting.

In addition to demographic data, participants were asked about the tools and strategies they use to transmit information within their teams. This included specific questions regarding the use of software applications (e.g., institutional email, instant messaging apps, shared documents, online surveys). Their responses provided detailed qualitative data on each tool’s frequency, purpose, perceived effectiveness, and limitations. These qualitative accounts were later grouped thematically during content analysis to assess patterns and preferences in software usage.

The interviews were conducted in locations and at times convenient for the participants.

All data collected was anonymous and strictly confidential, and the information obtained was used for research purposes only.

Interviews were audio-recorded and lasted, on average, 20 to 30 min, and were conducted by a nurse in the second year of the Master’s Degree of Science in Nursing, with previous training in qualitative research.

**Table 1:** Interview guide

Interview questions
1. *What kind of information do you share with your collaborators?*
2. *What strategies do you use to disseminate information to your collaborators?*
3. *How do you verify the actual transfer of information?*
4. *Can you recall a situation in which you recently shared some information?*
5. *Have you ever considered adopting alternative ways of disseminating internal communications? If so*,* which ones?*
6. *In your opinion*,* what are the factors that come into play in the process of dissemination of information?*
7. *Do you have any suggestions for managing the internal transfer of information within the hospital?*

To ensure data confidentiality, each interviewee was recorded and assigned an identification code. The verbatim transcripts of the interview audio recordings were assigned this code and can no longer be traced back to the interviewee.

### Data analysis

The interviews were transcribed verbatim by two authors independently. To ensure anonymity and confidentiality, participants were assigned identification codes during transcription.

An inductive qualitative content analysis approach was adopted, allowing categories to emerge directly from the data rather than from pre-existing theoretical frameworks [32]. Two authors independently coded the transcripts, and discrepancies were resolved through discussion with a third author to ensure rigor and consistency.

The analysis was iterative and consisted of several phases:

1. Familiarization with the data through repeated readings of the transcripts;
2. Identification of meaningful textual units relevant to the research question;
3. Assignment of descriptive labels to each unit of meaning;
4. Grouping of labels based on similarities to form categories.

The emerging categories and interpretations were reviewed by a third author to assess coherence between the data and the assigned labels. Field notes collected during interviews and analysis were also discussed, and the authors reflected on how their perspectives influenced interpretation.

Feedback from participants was sought to enhance the validity of the findings. All 19 participants were contacted and invited to review summaries of the emerging themes. Minor clarifications and adjustments were received and incorporated into the analysis to ensure that participants’ meanings and perspectives were accurately represented [33].

Throughout the study, a reflexive approach was adopted to minimize potential researcher bias. The research team consisted of nurses with experience in clinical practice and qualitative research within the same healthcare organization as the participants. Although the interviewers were not in a direct hierarchical relationship with participants, their professional background and organizational affiliation could have influenced data collection and interpretation. To mitigate this risk, interviews were conducted using open-ended questions and avoiding leading prompts. Coding decisions were documented, disagreements were resolved through consensus, and interpretations were regularly discussed among the research team to enhance reflexive awareness and analytic rigor.

No qualitative data analysis software was used. Manual coding was considered appropriate given the relatively small dataset and the iterative, team-based analytic process.

## Results

Nineteen Heads of Nursing and Allied Health Professionals participated in the study. Of the 19 participants, 14 were Heads of Nursing and 5 were Heads of Allied Health Professionals (two midwife managers, one physiotherapy manager, one laboratory technician manager, and one radiology technician manager). Most were female (73.7%) and had an average of 10.9 years of experience in their roles, ranging from 3 to 30 years. Participants interviewed belong to all areas: medical, surgical, emergency, diagnostic, and obstetrics-gynaecology (Table 2).

**Table 2:** Characteristics of participants

Characteristics of participants	*n*	%
Number of participants	19	
Age (years)		
35–50	8	42.1
51–65	11	57.9
Experience in their current positions (years)		
3–9	10	52.6
10–20	6	31.6
> 20	3	15.8
Gender		
Female	14	73.7
Male	5	26.3
Areas		
Medical	5	26.3
Surgical	5	26.3
Emergency-urgency	3	15.8
Obstetrics-gynaecology	3	15.8
Diagnostics	3	15.8

From the analysis of the interviews, five categories emerged regarding opinions and experiences concerning the internal information transmission process: use of software applications for the transmission of information; Time management for the transmission of information; Use of feedback as a verification strategy; contextualisation of information in the Care Unit; Lived with uncertainty about the effectiveness of official communication channels.

Although frequencies and percentages are reported for clarity, the analysis remains qualitative and interpretative, focusing on the depth and richness of participants’ perspectives rather than numerical generalization.

### Theme 1. Use of software applications for the transmission of information

#### Subtheme: Formal and informal digital communication channels

This theme encompasses both formal channels, such as institutional email and scheduled meetings, and informal channels, such as instant messaging apps. It illustrates how leaders adapt these tools to balance speed, clarity, and confidentiality in transmitting information.

Participants described their use of various software applications to transmit information, ranging from formal channels, such as email, to informal channels, such as messaging apps and social networks.

All the leaders interviewed disseminate information formally via institutional e-mail and scheduled meetings, with some also offering the option to participate remotely via multimedia platforms. This approach ensures that information has been effectively and correctly disseminated, thus enabling accurate tracking and documentation of communications.

…*we use the institutional email channels and the communication channel of meetings*,* also taking advantage of the technologies made available recently…*.

Many participants reported using instant messaging apps, mainly to draw attention to urgent emails sent through institutional channels. However, many of these leaders avoided using apps for transmitting detailed content in order to safeguard confidentiality.

…*when I have urgent emails*,* which I need to be viewed immediately*,* I write on the WhatsApp group “look at the email I sent you an urgent communication”*,* I use it in these ways…*.

However, a small proportion of those using instant messaging apps also share content information but do not use it as a preferred method, only for urgent information and always followed by communication through formal channels. This method is chosen for its speed and effectiveness in transmitting messages that require immediate attention while maintaining the formality and completeness of communication through the main institutional channels. This way, leaders can deal with critical situations promptly while ensuring that all official information is archived and accessible through formal channels.

…*it’s also been used a lot to give service announcements*,* although it’s not a medium that’s considered suitable… but maybe things at the last second… I wrote it on WhatsApp after I sent it in the email.*

In addition to instant messaging apps, this category includes software applications that allow people to answer online questionnaires and share editable documents.

*I also did a questionnaire on Google Drive with some answers that gave me an important overview of the level of information there is…also to know how to behave and where I need to focus.*

However, some of the interviewed state that they do not agree with the use of instant messaging apps for work purposes, as they believe that this channel, being unofficial, could invade the personal sphere of HCWs by creating a continuum even outside working hours.

… *WhatsApp means that a person never has the moment where they say ‘I’m done*,* I’ve clocked out*,* I’m home’*,* you’re always involved … that maybe it’s … a system that forces us never to disconnect.*

Participants reported limiting the use of instant messaging applications mainly due to concerns about privacy, confidentiality, and the lack of institutional recognition of these tools. Institutional email and scheduled meetings were perceived as offering greater traceability and accountability for formal communications, while informal channels were used cautiously and primarily for alerting purposes.

### Theme 2. Time management for the transmission of information

#### Subthemes: Organizing meetings, managing written information, information overload

This theme captures leaders’ experiences with time constraints and highlights three interconnected aspects: organizing dedicated meetings for communication, handling written information such as emails efficiently, and managing the overload of incoming information. These subthemes together illustrate how time management affects the clarity and effectiveness of top-down communication.

In the category “time management for the transmission of information”, all labels related to the interviewees’ perception of the lack of time for transmitting information were grouped.

Many participants emphasized the need to dedicate adequate time to organizing targeted meetings to transmit information. These meetings are crucial to communicating the necessary information and receiving immediate and contextual feedback from the participants. They believe that more effective time management would significantly improve the quality of internal communication. Organizing regular, dedicated information-sharing meetings would help transmit directives clearly and monitor and verify HCWs’ understanding and application of them.

…*I would need more time basically because we are overloaded with activities*,* so sometimes even checking whether information has been understood takes time…*

The interviews also reveal the leaders’ concern about ensuring adequate time spaces for HCWs to receive the information properly. These spaces, excluded from work activities, should allow HCWs to participate peacefully in meetings, thus improving the understanding and application of information.

…*it would be ideal to create or set up a time*,* outside of work activities*,* to have the serenity to meet*,* exchange*,* and share information.*

In addition to the time required to attend meetings, it was also pointed out that a considerable time commitment is required to handle emails. This includes not only the time to read them but also the time needed to understand their content, which can be particularly demanding due to the large number of messages and the complexity of the information. Moreover, dual-factor authentication for accessing the email application entails additional time commitments.

… *so*,* for email communication certainly the… workload*,* in my opinion*,* affects a lot… so if they during the shift… don’t have time to look at the email because they are too overloaded to look at it then they don’t look at it…*

The lack of time for transmitting information can be attributed to information overload, making it difficult to establish a degree of importance for each piece of information. This requires time management, as reading, understanding, and responding to each email requires concentration and attention. Not all emails are equally important; distinguishing between urgent and ordinary messages can be challenging. In addition, this information overload also affects HCWs, reducing the time available to provide direct patient care and diverting attention from important information.

*You have to consider that if I have too much information*,* even the most important information might slip through my fingers….*

### Theme 3. Use of feedback as a verification strategy

#### Subthemes: Verbal feedback and direct observation

This theme captures the strategies leaders use to verify that information has been received and understood. Two main subthemes emerged: requesting verbal feedback to confirm comprehension and using direct observation to monitor whether information is effectively applied in practice.

In all interviews, the use of feedback as a strategy for verifying the information received emerged. This practice includes various methods, from verbal feedback requests to direct observation, to assess the action’s adherence to what was requested.

Most interviewees stated that they verify the transmission of information by asking for verbal feedback, explicitly asking whether the information conveyed has been read and understood. This approach allows for immediate and direct feedback on the understanding and accepting the communicated information. Some interviewees also highlighted the usefulness of open-ended questions, which stimulate deeper reflection and offer a more comprehensive view of the level of understanding.

*It is not enough just to give the information*,* but to have feedback that the person has understood the information…*

In addition to verbal feedback, in some cases, interviewees use direct observation as a complementary method. They resort to direct observation of clinical practice or patient records to obtain feedback, depending on the information transmitted. This practice involves monitoring employees’ behaviour and actions to ensure they align with the information received. Direct observation makes it possible to identify discrepancies between the communicated information and the practical implementation, offering the opportunity to intervene promptly to correct any misunderstandings.

…*you try to get feedback in the field*,* see if things are put into practice…*

### Theme 4. Contextualisation of information in the care unit

#### Subthemes: Analysis and contextualization, content synthesis, prioritization of information

This theme explores how leaders adapt and contextualize information received from higher management to the specific needs of their units. Three subthemes emerged: analysing and reflecting on information to understand its implications, synthesizing, and simplifying content to make it actionable, and prioritizing the most relevant information for effective dissemination to HCWs.

Most participants report that personal insight and analysis of the information received are fundamental to optimizing its contextualization to the coordinated unit. Respondents emphasize that, in addition to receiving information, it is essential to spend time on reflection and analysis, which allows them to identify relevant details, understand the implications of the information, and evaluate how it integrates with the specific dynamics and requirements of their role and in their own unit.

…*there are things that come from the top to me as well*,* but maybe we can adapt them a little bit to our unit and then you just try to readapt them a little bit*,* that’s um… as far as you can.*

In addition to contextualizing information, many respondents reported synthesizing the information they receive, disseminating only what they consider to be key concepts, or reworking the original information to make it simpler, more immediate, and actionable. This synthesis and simplification process is essential to ensure that information is understood and used effectively within the organization.

*…* *I choose whether by default I have to disseminate it and forward it as it is to my staff or whether I can somehow synthesize it*,* modify it*,* to arrive at a communication that can be more immediate and synthesized.*

Finally, some interviewed leaders stated that they find it useful to establish an order of importance and relevance to the information they receive, even marking with alerts those they consider most important to facilitate the use of the content for HCWs

*…* *and also the placement in a priority order*,* because as we know*,* the space for the reception of information*,* which must be assimilated*,* is not infinite…*

### Theme 5. The uncertainty on the effectiveness of official communication channels

#### Subthemes: Technological and generational barriers, engagement in remote meetings

This theme reflects leaders’ experiences of uncertainty regarding whether information disseminated through official channels is actually received and understood. Two subthemes emerged: barriers related to technology use and generational differences among HCWs, and challenges in ensuring engagement during remote meetings.

Nearly all participants revealed an experience of uncertainty regarding the real effectiveness of disseminating information through formal channels, especially through institutional e-mails. The perception of the Head of Nursing and Allied Health Professionals is that emails are often not read or, at least, not read quickly.

*…I have noticed this difficulty in reading the e-mail; I don’t know what it is due to*,* but it is so…*

To deal with this problem, some leaders reported using email with read confirmation for communications they consider important. However, they found that read confirmations are not received if the emails are read from smartphones, that the HCW may decide not to send the read confirmation even when reading the email, and that, in any case, there is no certainty that the information is read even when the read confirmation is received.

*As for the emails*,* at least they opened them with the confirmation. But I don’t have the certainty that a person will read it; that’s why I told them*,* “This is your responsibility.”*

In some cases, the difficulty in using the applications by the HCWs belonging to the ‘Boomers’ (1946–1964) or ‘X’ (1965–1980) generations emerged as the reason for not reading the information transmitted via email.

Gen Y (1981–1996) may be more computer-savvy and technology-ready than the Boomers and Gen X and, therefore, more prepared to work with computing devices [34–36].

*Then it has to be said that not all of them know how to use computers because they are of a certain age they are reluctant*,* some are reluctant*,* they don’t know how to do it*,* they don’t even want to try*,* but it’s understandable because of their age.*

A small proportion of respondents also expressed concern that HCWs connected to remote meetings do not closely follow the information being transmitted.

…*now*,* instead of with the fact that you can have meetings with… You are in a meeting with maybe 10 people*,* and the other 25/30 work remotely from home; you don’t know if they are following everything; yes*,* maybe they are connected*,* but actually*,* you don’t know if they are there listening or not listening… or they are doing their own thing at home. So maybe they are not completely present at that moment.*

## Discussion

The findings of this study support the view that top-down communication functions as a sensemaking process. Participants described extensive efforts to interpret, prioritize, and contextualize information before forwarding it to staff, consistent with the role of middle managers as meaning-makers within complex organizations [25]. Rather than acting as passive conduits, leaders actively shaped the content, timing, and modality of communication to enhance comprehension and feasibility in their units.

The preference for specific communication channels observed in this study can also be interpreted through media richness theory, which posits that richer media are better suited for conveying complex or ambiguous information [26]. Additionally, reports of excessive communication and the need to filter messages align with literature on information overload, highlighting how high message volume can challenge leaders’ capacity to process and disseminate information effectively [27].

The analysis of the interviews revealed key aspects of the internal information transmission process. Institutional email emerged as the predominant formal channel, whereas instant messaging applications were used more selectively, primarily for urgent alerts rather than for detailed communication. Feedback was commonly described as a mechanism to support understanding, while only a few participants reported using direct observation to verify implementation. Overall, these findings indicate that internal communication in hospital care units is not merely a technical transmission of messages but a dynamic interpretive activity embedded in everyday managerial work. Leaders continuously negotiate between organizational demands for standardization and the practical need for flexibility, adapting communication strategies to situational constraints, urgency, and staff characteristics. From this perspective, top-down communication can be understood as a process of sensemaking rather than a linear transfer of information.

The use of software applications emerged as a central issue. French-Bravo et al. [22] showed that Heads of Nursing and Allied Health Professionals employ multiple dissemination methods, including e-mail, meetings, telephone calls, text messages, staff meetings, annual evaluations, and individual meetings. Similarly, participants described relying primarily on formal channels such as institutional e-mails and scheduled meetings, often supported by digital platforms. However, the use of instant messaging apps such as WhatsApp for urgent communications remains controversial. Most interviewees reported limiting their use to avoid privacy risks and protect work–life balance, reflecting a lack of formal institutional recognition. This highlights a tension between the efficiency of informal technologies and the need to maintain professional boundaries.

This tension can be interpreted as part of a broader organizational dilemma: while informal digital tools increase speed and reach, they may blur professional boundaries and shift responsibility for information management onto individuals. Leaders appear to adopt a gatekeeping role, preserving institutional channels as the legitimate space for formal communication while selectively using informal tools to compensate for system limitations. This dual-channel approach may therefore represent an adaptive strategy to organizational complexity rather than inconsistency in leadership practice.

Technology is recognized as a key factor influencing employee health and work stress [37, 38]. In Italy, the use of instant messaging apps at work is not governed by specific legislation but is subject to privacy principles, the right to disconnect, and confidentiality rules, particularly under GDPR and the Italian Criminal Code. Employers cannot access private communications for disciplinary purposes, and employees have the right not to be contacted outside working hours. Consistent with this regulatory context, participants reported that instant messaging apps were rarely used to transmit detailed content, but rather to draw attention to urgent information delivered through formal channels such as e-mail. Institutional email and meetings were perceived as more appropriate for formal and sensitive information due to traceability and confidentiality.

At the same time, leaders expressed uncertainty about whether institutional communications were actually read or implemented. The healthcare work environment is characterized by variability and complexity [21], and the volume of available information has become excessive. Information overload is recognized as a major stressor for healthcare workers [39, 40]. Our findings suggest that difficulties arise not only from the quantity of information but also from its complexity and the time required to manage it. These challenges may reduce the time available for patient care and increase the risk that important messages are overlooked.

Generational differences in the use of digital tools also emerged. Failure to read institutional emails was sometimes attributed to difficulties with digital technologies amongs Boomers generation (1946–1964) and Generation X (1965–1980) whereas younger professionals appeared more comfortable with digital communication. This is consistent with literature indicating that Generation Y (1981–1996) tends to be more computer-literate and technologically ready than previous generations, and therefore more confident in working with digital devices [34–36]. However, generational factors did not operate in isolation. Participants emphasized that workload, time pressure, and unit culture often influenced channel choice across all age groups. In highly demanding clinical environments, instant messaging was frequently preferred because of its speed, whereas units with structured routines tended to rely more on formal channels.

These findings suggest that technology use in internal communication is shaped less by individual competence alone and more by organizational context. Unit culture, leadership expectations, perceived urgency, and staffing levels influence whether digital tools complement or substitute official channels. Viewing technology use as an organizational capability rather than an individual skill provides a more nuanced understanding of communication practices in healthcare settings.

The literature highlights leaders’ efforts to understand barriers by actively seeking feedback and asking “why,” thereby facilitating engagement with healthcare workers [22]. Similarly, participants described requesting feedback verbally or by e-mail and, in some cases, using direct observation of clinical practice or documentation to verify effectiveness. Leaders also emphasized the need to synthesize and contextualize information, adapting messages to the realities of the care unit. This aligns with recommendations that information should be concise, context-sensitive, and clearly understood by recipients to prevent miscommunication and inefficiencies [10, 41, 42].

An increasing body of research examines how information and communication technologies influence healthcare systems [43]. Consistent with previous studies, successful message delivery does not guarantee that messages are read or understood [10, 44]. Leaders therefore reported preferring face-to-face communication or telephone contact for high-priority issues, as these methods allow real-time clarification and feedback. Despite technological advances, direct interpersonal communication remains highly valued but is often constrained by time and workload.

Some issues emerged only partially and warrant further investigation. Group involvement appears to influence how information is disseminated and received, and individual factors such as stress and motivation may affect comprehension. High work stress can impair the ability to process information effectively [45], increasing the risk of communication breakdowns.

Leaders face the ongoing challenge of selecting communication methods that ensure messages are both received and understood. Although text messaging is convenient, excessive volume may reduce effectiveness. Therefore, digital tools should complement rather than replace established communication practices [10]. Combining multiple channels and tailoring messages to specific contexts may enhance engagement and effectiveness [46–48]. Participants reported using several strategies simultaneously — including calls, texts, emails, meetings, and noticeboards — to reach staff working different shifts and ensure consistent information dissemination [44].

These findings provide a basis for developing strategies to improve communication and information management in healthcare organizations. While this study focused on communication between leaders and staff, the growing emphasis on patient and family engagement suggests the need to explore how nurse managers communicate beyond professional teams. Future research could examine strategies to support understanding, involvement, and shared decision-making.

More broadly, the results indicate that effective internal communication should be conceptualized as a multi-layered organizational process integrating technical systems, leadership judgment, and relational work. Rather than identifying a single “best” channel, communication effectiveness depends on leaders’ ability to select, combine, and adapt strategies to situational demands. Further investigation of these dynamics could support the development of more effective solutions for everyday operational challenges.

## Study limitations

This study presents some limitations inherent to its qualitative methodology and specific context. The research was based on a relatively small sample of Heads of Nursing and Allied Health Professionals in a single University Hospital in Northern Italy, limiting the generalisability of the results to other healthcare facilities. Additionally, using non-probabilistic sampling methods, such as purposive sampling, further impacts generalisability. However, it is important to note that in qualitative research, the aim is not statistical generalisability but theoretical generalisability, which is the potential to transfer findings to contexts similar to the one studied [49].

Moreover, the qualitative nature of the interviews introduces potential interpretative biases resulting from the personal experiences and perceptions of both researchers and interviewees, although feedback mechanisms partially mitigate this risk.

Lastly, the study exclusively focused on leaders, excluding the perspective of HCWs who receive communication. As a result, the findings might be biased, as they consider only one of the two parties involved in the communication process and primarily reflect managerial interpretations of communication effectiveness. The perspectives of frontline HCWs may differ, particularly regarding clarity, accessibility, and usability of information, and were not captured in this study. Future research should therefore include multiple organizational levels to provide a more comprehensive and balanced understanding of internal communication processes.

Another limitation is the absence of participants under 35 years of age. This may have influenced the results, as younger nurse managers might have different attitudes toward digital communication and hierarchical relationships compared to their older colleagues.

## Conclusion

The analysis of interviews highlights the complexity of internal information transmission in healthcare organizations. The five main categories identified provide a comprehensive view of the challenges and strategies leaders adopt to improve organizational communication. They also require integrated strategies to balance technological efficiency, HCWs’ welfare, and care quality.

Challenges such as the use of software applications, information overload, and balancing formal and informal communication methods underscore the need for a multifaceted approach. Integrating multiple communication channels, contextualizing information, and prioritizing face-to-face interactions can enhance clarity, trust, and message comprehension.

Future studies should explore the perspectives of healthcare workers, group involvement, and the impact of organizational climate on communication to develop more tailored and effective solutions.

In conclusion, an integrated communication approach that balances traditional and informal methods, manages information overload, and dedicates time to communication is vital for improving efficiency and quality.

## Data Availability

The datasets generated and/or analysed during the current study are not publicly available due to confidentiality agreements with the participants but are available from the corresponding author on reasonable request.

## Abbreviations

HCWs: Healthcare workers
GDPR: General Data Protection Regulation
Gen X: Generation X
Gen Y: Generation Y
